# Circulating biomarkers of infection and endometrial cancer risk

**DOI:** 10.1007/s10552-025-02080-6

**Published:** 2025-10-15

**Authors:** Kara A. Michels, David Jin, Rima Jeske, Jolanta Lissowska, Beata Pepłońska, Nicolas Wentzensen, Tim Waterboer, Britton Trabert

**Affiliations:** 1 Division of Cancer Epidemiology and Genetics, National Cancer Institute, National Institutes of Health, Rockville, MD USA; 2 Department of Epidemiology and Population Health, Albert Einstein College of Medicine, Bronx, NY USA; 3 Division of Infections and Cancer Epidemiology, German Cancer Research Center, Heidelberg, Germany; 4 Department of Cancer Epidemiology and Prevention, Maria Sklodowska-Curie National Research Institute of Oncology, Warsaw, Poland; 5 Department of Environmental Epidemiology, Nofer Institute of Occupational Medicine, Łódź, Poland; 6 Department of Obstetrics and Gynecology, University of Utah, Huntsman Cancer Institute at the University of Utah, Salt Lake City, UT USA

**Keywords:** Endometrial neoplasms, Chlamydia trachomatis, Herpesvirus 2, Human, Molecular epidemiology, Histology

## Abstract

**Purpose:**

Incidence and mortality rates for endometrial cancer are rising. We need to better understand the etiology of this disease and identify new risk factors. We examined whether common genital infections are associated with endometrial cancer.

**Methods:**

Using serum samples from The Polish Endometrial Cancer Study (443 cases, 443 controls), we measured antibodies against microbial antigens with a multiplex fluorescent bead-based assay. We estimated adjusted odds ratios (OR) and 95% confidence intervals (CI), comparing women with positive versus negative serology.

**Results:**

Most antibodies were not associated with endometrial cancer overall, but seropositivity for *Herpes simplex virus 2* was associated with low-grade tumors (OR 1.43 CI 1.02, 2.00). While increased risks for type II, but not type I tumors, were consistently indicated for multiple *Chlamydia trachomatis* antigens, most estimates did not reach statistical significance (e.g., Pgp3 seropositivity and type I cancers: OR 0.97 CI 0.73, 1.31; and type II: OR 2.96 CI 0.85, 10.31; heterogeneity *p*-value = 0.03). The strongest associations for type II tumors were observed with seropositivity for *Chlamydial* stress response proteins.

**Conclusion:**

Reproductive tract infections may increase risk for endometrial cancer, but the biologic mechanisms are likely both microbe- and histology-specific. Some *C. trachomatis* infections may be a risk factor for type II endometrial cancers. Given that so few risk factors for type II endometrial cancers are identified, infection-related mechanisms of carcinogenesis in the endometrium merit continued investigation.

**Supplementary Information:**

The online version contains supplementary material available at 10.1007/s10552-025-02080-6.

## Introduction

In many countries, incidence and mortality rates for endometrial cancer have risen dramatically in the last twenty years [1]. The majority of endometrial cancers are often referred to as type I tumors; they have an endometrioid or adenocarcinoma histologic subtype (histotype) [2]. These tumors are often identified at early stages because many women will present to their physicians with postmenopausal vaginal bleeding, though only a small proportion of women with bleeding will have endometrial cancer [3]. Women who develop non-endometrioid endometrial cancers are often categorized together in research analyses because these tumors are comparatively, less common than the endometrioid subtype. Type II endometrial cancers include serous or clear cell histotypes. Women diagnosed with non-endometrioid tumors have a worse prognosis than women diagnosed with endometrioid tumors [4]. Within the United States, the incidence rates for non-endometrioid cancers have increased by approximately 3% every year between 2000 and 2015 (versus 0.5% for endometrioid tumors) [4].

The associations between endometrial cancer and factors such as parity, oral contraceptive use, smoking, and diabetes, are similar by tumor histotype [5]. Obesity is a stronger risk factor for low-grade endometrioid tumors compared to high grade endometrioid or type II tumors [5, 6]. This suggests that rising obesity rates do not fully account for the increasing incidence in non-endometrioid tumors, relative to endometrioid tumors. We need to improve our etiologic understanding of endometrial cancers and identify modifiable risk factors associated with this disease.

Research indicates that infections, such as those caused by *Chlamydia trachomatis*, may increase risk for ovarian cancer [7–11], but microbial exposures are not well-studied as risk factors for endometrial cancer. To this end, we wanted to understand if endometrial cancer is associated with exposure to infections that can potentially ascend the reproductive tract. Using data from the Polish Endometrial Cancer Study, we examined endometrial cancer risks associated with circulating antibodies specific for antigens from a range of microbes, including *C. trachomatis* and *Herpes simplex virus 2* (*HSV-2*).

## Materials and methods

### Study population

The Polish Cancer Study was a large case–control study of endometrial, ovarian, and breast cancer that enrolled women aged 20–74 years from Warsaw or Łódź, Poland [12, 13]. The women in the endometrial cancer study were enrolled between 2001 and 2003 [12]. Women with incident and pathologically confirmed endometrial cancer were identified through a hospital-based rapid identification system that covered approximately 85% of all cancer cases in Warsaw and Łódź; cancer registries were used to identify additional women with cancer for potential recruitment into the study [14]. To serve as population-based controls, women were selected from the Polish Electronic System, a database with demographic information for all residents of Poland [12].The control group was frequency matched to cases on study site and age group, and was limited to women with intact uteri and without a history of breast or endometrial cancer [14]. In-person interviews were conducted to obtain information on demographics, anthropometric factors, body mass index, health history, and lifestyle behaviors at the time of study enrollment; blood samples were also collected from participants. Protocols for the Polish Endometrial Cancer Study were approved by local institutional review boards and the U.S. National Cancer Institute [12]. All participants provided written informed consent.

### Multiplex serology

We measured antibodies against antigens from *C. trachomatis*, including the commonly studied plasmid-encoded virulence factor Pgp3, major outer membrane proteins (MOMP), translocated actin-recruiting phosphoprotein (Tarp, divided into C- and N- terminal fragments), and heat shock protein 60 variant 1 (HSP60-1); the other antigens from *C. trachomatis* are named for their gene sequence and may encode proteins with known or unknown function. We also assessed antigens from microbes known to cause reproductive tract pathologies, including *Mycoplasma genitalium* (MgPaN and rMgPa, antigens from two regions of the adhesin P1), *HSV-2* (mgG, mucin-like glycoprotein), and *Human Papilloma viruses* (HPV) 16 and 18 (major capsid proteins L1), as well as antigens from *Polyomaviruses BK*, *JC*, and *6* (VP1, viral capsid protein 1) as positive controls (i.e., we expected seropositivity to be high, but no association with cancer).

Serum samples were sent to the German Cancer Research Center (Heidelberg, Germany) for the assessment of antibody seropositivity to selected microbial antigens. The methods for the multiplex serology assay have been previously described [7, 15]. In brief, the assay is a bead-based suspension array in which selected antigens are expressed as recombinant glutathione S-transferase fusion proteins in *Escherichia coli*; the proteins are then loaded onto fluorescently labeled polystyrene beads (Luminex Corp., Austin, Texas, USA) derivatized with glutathione-casein. Study serum samples were incubated with the beads at a final 1:100 dilution. Biotinylated goat anti-human IgG/IgM/IgA secondary antibodies (Jackson ImmunoResearch, West Grove, Pennsylvania, USA) and Streptavidin-R-Phycoerythrin (MossBio, Pasadena, MD, USA) were used to quantify human antibodies bound to the beads. The final fluorescence corresponded to the amount of serum antibodies (i.e., “antibody levels”) and was measured in units of median fluorescence intensity (MFI) on a Luminex 200 analyzer. Final MFI values were estimated by subtracting the MFI of a serum-specific GST control and the bead-specific background fluorescence from the raw MFI values [15]. Continuous final MFI values were dichotomized as seropositive or seronegative based on previously used cut-points, which were adjusted to reflect the measured signal distribution [7, 16]. Three serum pools were included in duplicate across batches as quality control samples. The average concordance of seropositivity status between these samples was 92.6% across all antigens (median = 93.6%, standard deviation = 7.9%, minimum = 71.%, maximum = 100%).

### Statistical analyses

We used adjusted conditional logistic regression to estimate odds ratios (OR) and 95% confidence intervals (CI) for endometrial cancer, comparing women with positive versus negative serology for antibodies against each antigen. The main models were conditioned on matching factors (age and study site) and then adjusted for history of oral contraceptive use (never/ever), and current smoking (never, past, current), body mass index (< 25, 25–29.9, 30 + kg/m^2^), and menopausal status (premenopausal, postmenopausal).

In secondary analyses, we evaluated associations by endometrial tumor type (I and II) and grade (low: well or moderately differentiated, high: poorly differentiated). In these analyses, we used unconditional multinomial logistic regression adjusted for the aforementioned matching factors and variables, with controls as the reference group. For the tumor type analyses, we also ran cases-only models comparing women with type II tumors to type I tumors (as the reference group) to estimate p values for heterogeneity (p-het) from Wald tests for each antigen of interest. Data missingness was minimal and a complete-case modeling approach was used. An α of 0.05 was used to estimate statistical significance, all statistical tests were two-sided, and analyses were performed using SAS (SAS Institute, Cary, NC).

## Results

Basic characteristics of the participants in the Polish Endometrial Cancer study are described in Table 1. When considering all endometrial cancer types together, our data suggest that most antibodies were not associated with endometrial cancer (Table 2; Supplemental Table S1). An increased risk of borderline statistical significance for any endometrial cancer was indicated with seropositivity against the *C. trachomatis* antigen, CT_418 (odds ratio [OR] 1.79 and 95% confidence interval [CI] 0.96, 3.23). A modest association was suggested for seropositivity against the *HSV-2* antigen, mgG (OR 1.21 CI 0.87, 1.69). Positive serology for *Polyomavirus JC* was associated with a reduced risk for endometrial cancer, but most women in this population (75–97%) were expectedly seropositive for the virus.

**Table 1:** Selected characteristics of cases and controls from the Polish Endometrial Cancer Study

	Cases *n* = 443		Controls *n* = 443	
	*n*^a^	%	*n*	%
Age category in 5-yr intervals				
< 35	3	0.7	3	0.7
35–39	2	0.5	2	0.5
40–44	9	2.0	9	2.0
45–49	33	7.5	33	7.5
50–54	55	12.4	55	12.4
55–59	82	18.5	82	18.5
60–64	86	19.4	86	19.4
65–69	95	21.4	95	21.4
70 +	78	17.6	78	17.6
Body Mass Index (kg/m^2^)				
< 25	118	26.6	155	35.0
25–29.9	167	37.7	173	39.1
30 +	153	34.5	108	24.4
Menopausal Status				
Premenopausal	6	1.4	69	15.6
Postmenopausal	437	98.7	373	84.2
Highest education level attained				
Did not complete high school	157	35.7	178	40.3
High school graduate	160	36.4	155	35.1
Some college, professional training	32	7.3	40	9.1
College graduate	91	20.7	69	15.6
Smoking status				
Never	284	64.1	224	50.6
Past	79	17.8	90	20.3
Current	80	18.1	129	29.1
Oral contraceptive use				
Never	420	95.2	415	94.5
Ever	21	4.8	24	5.5

^a^Columns and percentages may not sum to total due to missing data; less than 2% of the population were missing data on any of these variables

**Table 2:** Associations between seropositivity against selected microbial antigens and endometrial cancer

*Microbe* and Antigen	Seropositivity				Association with endometrial cancer	
	Cases		Controls		OR^b^	95% CI
	*n* + ^a^	% +	*n* +	% +		
*Chlamydia trachomatis*						
Pgp3	184	41.5	181	40.9	1.11	0.82, 1.50
MOMP-A	158	35.7	159	35.9	0.96	0.70, 1.32
MOMP-D	175	39.5	183	41.3	0.98	0.72, 1.35
MOMP-L2	156	35.2	164	37.0	1.10	0.79, 1.54
Tarp-F1	203	45.8	212	47.9	0.88	0.65, 1.19
Tarp-F2	230	51.9	213	48.1	1.16	0.85, 1.59
HSP60-1	236	53.3	228	51.5	1.13	0.82, 1.56
CT_067 (TroA)	152	34.3	149	33.6	1.04	0.74, 1.46
CT_223	207	46.7	202	45.6	1.00	0.73, 1.35
CT_418	39	8.8	25	5.6	1.76	0.96, 3.23
CT_664C	197	44.5	188	42.4	1.20	0.88, 1.65
CT_664N	137	30.9	133	30.0	1.08	0.77, 1.52
CT_798	191	43.1	184	41.5	1.08	0.80, 1.46
*Mycoplasma genitalium*						
MgPaN	204	46.1	216	48.8	0.91	0.66, 1.26
rMgPa	250	56.4	257	58.0	1.02	0.74, 1.40
*Herpes simplex virus 2*						
mgG	160	36.1	137	30.9	1.21	0.87, 1.69
*Human Papilloma virus*						
16L1	22	5.0	23	5.2	0.81	0.41, 1.60
18L1	43	9.7	44	9.9	0.99	0.60, 1.63
*Polyomavirus*						
BKVP1	433	97.7	432	97.5	1.29	0.42, 3.97
JCVP1	333	75.2	361	81.5	0.62	0.42, 0.92
HPyV6VP1	412	93.0	398	89.8	1.62	0.86, 3.06

^a^Seropositive (+)

^b^Logistic regression models conditioned on matching factors (age and study site) and adjusted for oral contraceptive use, smoking, body mass index, and menopausal status

Four hundred thirty one women were diagnosed with type I endometrial cancers, while 12 women were diagnosed with type II tumors (Table 3 and Supplementary Table S1). The effect magnitudes for *HSV-2* seropositivity were comparable between type I (OR 1.26 CI 0.93, 1.71) and type II tumors (OR 1.40 CI 0.42, 4.06; p for heterogeneity 0.760). *HSV-2* seropositivity was associated with risk for low-grade tumors (OR 1.43 CI 1.02, 2.00; *n* = 291; not tabulated).

**Table 3:** Seropositivity against selected *Chlamydia trachomatis* and *Herpes simplex virus 2* antigens and endometrial cancer risk, by classical tumor type

*Microbe* and Antigen	Tumor Type						P-het
	Type 1^a^ *n* = 431			Type 2 *n* = 12			
	Cases *n* + ^b^	OR	95% CI^c^	Cases *n* +	OR	95% CI	
*Chlamydia trachomatis*							
Pgp3^d^	176	0.97	0.73, 1.31	8	2.96	0.85, 10.31	**0.031**
MOMP-A	153	0.96	0.71, 1.30	5	1.30	0.39, 4.36	0.635
MOMP-D	169	0.89	0.66, 1.19	6	1.45	0.45, 4.70	0.368
MOMP-L2	149	0.91	0.67, 1.23	7	2.69	0.81, 8.93	**0.050**
Tarp-F1	195	0.79	0.59, 1.06	8	1.94	0.56, 6.67	0.110
Tarp-F2	221	1.06	0.79, 1.42	9	3.39	0.88, 13.01	**0.073**
HSP60-1	230	1.09	0.81, 1.45	6	1.10	0.33, 3.66	0.906
CT_067 (TroA)	145	0.96	0.71, 1.30	7	2.78	0.84, 9.25	**0.052**
CT_223	198	0.93	0.70, 1.24	9	3.29	0.86, 12.56	**0.034**
CT_418	36	1.50	0.84, 2.68	3	**7.90**	**1.83, 34.05**	**0.024**
CT_664C	192	1.26	0.93, 1.69	5	1.25	0.38, 4.17	0.994
CT_664N	131	1.04	0.76, 1.42	6	3.13	0.92, 10.63	**0.060**
CT_798	183	0.94	0.70, 1.25	8	2.47	0.71, 8.56	**0.097**
*Herpes simplex virus 2*							
mgG	155	1.26	0.93, 1.71	5	1.40	0.42, 4.06	0.760

^a^Type I tumors include those with an endometrioid, mucinous, malignant mixed Mullerian, or Mixed histologic subtype. Type II tumors include those with clear cell and papillary serous histologic subtypes

^b^ Number of women with cancer who were seropositive (+)

^c^ Adjusted for age, study site, oral contraceptive use, smoking, body mass index, and menopausal status

^d^Statistically significant results are in bold. P-het in bold suggest potential type-specific associations, as defined by p for heterogeneity < 0.10

While modest risks for type I cancers were suggested with seropositivity against the *C. trachomatis* antigens CT_418 and CT_664C, null associations could not be ruled out (Table 3). Interestingly, the magnitude of the associations for multiple *C. trachomatis* antigens, including Pgp3, qualitatively differed by tumor type (Pgp3 seropositivity, type I: OR 0.97 CI 0.73, 1.31; and type II: OR 2.96 CI 0.85, 10.31; p-het 0.031). Across the *C. trachomatis* antigens evaluated, we consistently observed potential 2- to eightfold increases in the ORs for type II endometrial cancers, while ORs for type I cancers were near-null in magnitude—though the confidence intervals for only one of the type II associations reached statistical significance (p-het < 0.10). The strongest association we identified for type II tumors was with CT_418 seropositivity (OR 7.90 CI 1.83, 34.05; p-het 0.024).

## Discussion

To our knowledge, this is the first study to report on associations between endometrial cancer and exposure to *Chlamydia trachomatis*. Increased risks for type II tumors were suggested with antibody seropositivity to several *C. trachomatis* antigens; an eightfold increased risk associated with the CT_418 antigen reached statistical significance. In our study, most women diagnosed with type II cancers had serous tumors, but due to small sample sizes, we were unable to evaluate associations with the serous histotype alone—therefore, these results require replication and expansion. Although some of our estimates were not statistically significant, the consistency in the direction of the observed associations across chlamydial antigens supports the idea that chlamydia infection may be a promising avenue of etiologic research for endometrial cancer.

Seropositivity to *C. trachomatis* has been associated with increased risk for ovarian cancer in multiple studies, but the risks vary by population, tumor histotype, and the antigen of interest [7–11]. Most of these studies only evaluate seropositivity against a few antigens like Pgp3 and HSP60-1. We previously used data from the Polish Ovarian Cancer Study and a nested case–control study in The Prostate, Lung, Colorectal, and Ovarian Cancer Screening Trial (PLCO) to evaluate ovarian cancer risk associated with seropositivity to multiple *C. trachomatis* antigens, though we did not assess all of the antigens of interest from the present analysis (e.g., CT_418) [7]. In the data from Poland, the highest circulating levels of antibodies against Pgp3, MOMP-A, and MOMP-L2, were associated with ovarian cancer (ORs between 1.45 and 1.63) [7]. Similar effect magnitudes were observed within PLCO when higher Poland Study-based thresholds were used to determine seropositivity [7]. Interestingly, risks associated with Pgp3 were similar across ovarian cancer histotypes in the Poland data (serous: OR 2.24 CI 1.12, 4.46 versus non-serous: OR 2.06 CI 1.04, 4.08) [7].

We have not identified other studies reporting on *HSV-2* and endometrial cancer risk, though in a 1992 case series, researchers detected circulating *Herpesvirus* antibodies (type not specified) in 13 out of 20 women with endometrial cancer (most of whom had adenocarcinomas) [17]. *HSV-2* exposure does not appear to be a strong risk factor for ovarian cancer, but any associations may depend on other infections or tumor histotype. For example, results from the Nurse’s Health Studies I and II indicated that *C. trachomatis* infection was associated ovarian cancer (risk ratio [RR] 2.07 CI 1.25–3.43), while *HSV-2* infection was not (RR 1.38 CI 0.79, 2.42) [11]. However, when examining seropositivity to these pathogens together, ovarian cancer risks were strongest among women exposed to both *C. trachomatis* and *HSV-2* (reference group: unexposed women; only Pgp3 seropositive: RR 1.67 CI 0.97, 2.87; only *HSV-2* seropositive: RR 0.73 CI 0.33, 1.62; both Pgp3 and *HSV-2* seropositive: RR 3.26 CI 1.25, 8.51) [11]. A study from the European Prospective Investigation into Cancer and Nutrition cohort reported an association between *HSV-2* infection and endometrioid, but not serous, ovarian cancers (RR for endometrioid ovarian cancer: 2.35 CI 1.24, 4.43) [9].

It is important to remember that the Poland Endometrial Cancer Study was conducted over twenty years ago, before the recently observed increases in the incidence of endometrial cancer, especially in the United States [4]. We are not able to make direct connections between chlamydia infections and these trends with our data. This being said, the biologic plausibility for *C. trachomatis* increasing cancer risk merits discussion. The bacterium is known to modulate multiple cancer-associated metabolic pathways in host cells, including those involving tumor protein p53 and phosphatidylinositol 3-kinases (i.e., PI3K) [18]. Aberrant p53 staining and mutations in the p53 gene are a hallmark of serous endometrial cancers [2]. Pelvic inflammatory disease (PID) is also hypothesized to serve as a potential mechanistic link between reproductive tract infections and gynecologic cancers. Most PID is presumed to be caused by *C. trachomatis* or *Neisseria gonorrhea*, but contributions from other pathogens are increasingly being recognized [19, 20]. As such, this “inflammatory state” actually represents a range of heterogeneous exposures. Two research groups have explored associations between medical record documentation of PID and endometrial cancer, with both concluding that PID is not associated—but interpreting these results is difficult, given that the modeling strategy in one study only accounted for age and calendar period, while models in the other study were arguably overadjusted for comorbid conditions [21, 22].

The unique antigens that we evaluated also inform potential mechanistic links between chlamydia infection and cancer. For example, (TroA/YtgA) is a protein that may enable *C. trachomatis* survival in the face of environmental iron depletion; it is known to be expressed across multiple serovars [23, 24]. Our strongest association for type II endometrial cancers was observed with CT_418, though it should be noted that while 9% of all women with endometrial cancer in our study were seropositive for this antigen, only three women with type II tumors were seropositive. We are not aware of any other studies that have evaluated the seroprevalence of this antigen. This protein a member of the OGB superfamily of GTPases encoded by the gene *yhbZ* [25]. Increased transcription of the *obg* gene, though not necessarily translation, may be integral to stress responses across chlamydial species, especially when confronted with tryptophan depletion mediated by host interferon gamma secretion [26, 27]. In response to interferon gamma, intracellular *C. trachomatis* enters into a non-infectious, but persistent and metabolically active form (called a reticulate body); this state is also correlated with decreased levels of MOMP [28]. Taken together, the research on these antigens suggests that specific metabolic mechanisms enabling *C. trachomatis* persistence should be investigated in the context of gynecologic carcinogenesis—and for endometrial cancer, our analysis has demonstrated the importance of examining these potential connections across tumor histotypes.

We must acknowledge, while most of the antigens we evaluated are expected to be *C. trachomatis* derived, HSP60 for example, is a highly conserved protein across *Chlamydia* species [29]. Similarly, most of our antigens are likely produced across the serovars of *C. trachomatis*, which are defined by their unique major outer membrane proteins (MOMPs). Serovars A through C are associated with ocular chlamydial infections, serovars D through K cause genital infections, and serovars L1 through L3 cause lymphogranuloma venereum, but there can be overlap [30, 31]. Many of the chlamydial antigens selected for our multiplex assay were identified from a whole proteome array of serovar D [32], and for MOMP, we additionally evaluated seropositivity to the serovar-specific homologs of these proteins. The strongest MOMP association that we identified was between MOMP-L2 and type II endometrial cancers. Seropositivity to Pgp3, one of the most commonly used metrics for assessing prior *C. trachomatis* exposure, is also used in studies of ocular infection (trachoma) [33]. The Pgp3 protein is expressed across *C. trachomatis* strains, though the level of transcription will vary based on a strain’s propensity for infecting specific tissue types [34]. It is possible that variation in the prevalence of *C. trachomatis* infections by serovar contributes to disparate findings across study populations with regard to ovarian cancer risk associated with Pgp3.

A strength of our study is the use of circulating antibodies to measure infection history. Circulating antibodies are a reliable and informative way to assess infectious exposures in research settings. Antibodies are markers for specific microbes, and seropositivity is a metric that does not depend upon recall of infections (which can be asymptomatic) or upon subjective definitions of behaviors that might be used as proxies for infectious exposures (e.g., querying sexual history to assess sexually transmitted infection exposures). However, circulating antibodies are less useful for determining when an infection occurred, as women may have higher antibodies levels due to more recent infections or more severe infections (or perhaps, persistent infections). The extent to which circulating antibody concentrations wane over time is difficult to assess and likely dependent upon many factors, but Pgp3 seropositivity has been observed to persist for ten or more years in one study of reproductive aged women (with 97% of seropositive 26 year olds still being seropositive at age 38) [35]. We previously noted that *C. trachomatis* antibody levels were higher in the Poland Ovarian Cancer Study, relative to the older participants in PLCO [7]. If future studies can establish seropositivity cut-points that differentiate between recent or persistent infections, which we were not able to do, the latency between chlamydia infection and potential oncogenesis could be better understood.

From a clinical perspective, the odds ratio magnitudes we observed can be considered modest; these associations imply that the measurement of antibody seropositivity alone would not offer utility for endometrial cancer risk screening [36]. Additionally, we did not correct our findings for multiple testing because this was a hypothesis-generating study, so larger studies with more power will help confirm these exploratory results. However, our findings should inform etiologic investigations of endometrial cancer. Given that so few risk factors for serous endometrial cancers are identified [5], our study supports the need for continued investigation of reproductive tract infections as risk factors for this disease, particularly those stemming from *C. trachomatis*.

## Supplementary Information

Below is the link to the electronic supplementary material.

Supplementary file1 (DOCX 18 KB)

## Data Availability

Data from this study will be shared upon request. Please contact Nicolas Wentzensen for more information: [wentzenn@mail.nih.gov].
