# Supplementary material for: Circulating biomarkers of infection and endometrial cancer risk
Source: Cancer Causes Control. 2025 Oct 15;36(12):2015–22. doi: 10.1007/s10552-025-02080-6 (PMC12630196; doi:10.1007/s10552-025-02080-6)
Supplement: Supplementary file 1 — Supplementary file1 (DOCX 18 KB) [file 10552_2025_2080_MOESM1_ESM.docx]

**SUPPLEMENTARY MATERIAL**

**Supplementary Table S1. Associations between seropositivity against other evaluated antigens and endometrial cancer (not presented in main manuscript tables)**

| ***Microbe* and Antigen** | **Any endometrial cancer** | | **Type I^c^** | | **Type II^c^** | |
| --- | --- | --- | --- | --- | --- | --- |
|  |  |  |  |  |  |  |
|  | **OR^b^** | **95% CI** | **OR^b^** | **95% CI** | **OR^b^** | **95% CI** |
| ***Chlamydia trachomatis*** |  |  |  |  |  |  |
| CT_823 (HTRA) | 1.10 | 0.80, 1.51 | 1.08 | 0.81, 1.44 | 1.53 | 0.47, 4.99 |
| CT_051 | 0.87 | 0.64, 1.19 | 0.88 | 0.66, 1.18 | 0.55 | 0.16, 1.91 |
| CT_117 | 1.00 | 0.71, 1.41 | 0.97 | 0.70, 1.36 | 2.15 | 0.64, 7.21 |
| CT_142 | 1.08 | 0.79, 1.47 | 0.96 | 0.72, 1.28 | 1.54 | 0.47, 5.08 |
| CT_143 | 0.86 | 0.62, 1.21 | 0.86 | 0.63, 1.17 | 1.47 | 0.38, 5.70 |
| CT_187 | 1.02 | 0.64, 1.63 | 1.01 | 0.64, 1.59 | 0.85 | 0.10, 6.95 |
| CT_277 | 0.93 | 0.48, 1.78 | 1.04 | 0.55, 1.96 | 1.47 | 0.17, 12.32 |
| CT_496.1 | 1.26 | 0.60, 2.64 | 1.07 | 0.52, 2.23 | 2.85 | 0.32, 25.79 |
| CT_529 | 1.11 | 0.74, 1.65 | 0.95 | 0.66, 1.37 | 2.17 | 0.61, 7.64 |
| CT_576 | 0.96 | 0.69, 1.33 | 1.08 | 0.80, 1.46 | 0.94 | 0.27, 3.25 |
| CT_530 | 0.88 | 0.61, 1.29 | 0.94 | 0.66, 1.35 | 0.36 | 0.05, 2.89 |
| CT_707 | 1.11 | 0.70, 1.75 | 1.06 | 0.69, 1.63 | Could not be estimated | |
| CT_716 | 1.18 | 0.84, 1.65 | 1.11 | 0.81, 1.50 | 0.37 | 0.08, 1.74 |
| CT_813 | 1.02 | 0.71, 1.48 | 0.88 | 0.63, 1.24 | 0.95 | 0.24, 3.68 |
| CT_829 | 1.20 | 0.62, 2.34 | 1.16 | 0.62, 2.14 | 1.61 | 0.19, 13.76 |
| CT_847 | 1.02 | 0.69, 1.51 | 0.99 | 0.68, 1.45 | 1.12 | 0.23, 5.41 |
| CT_858N | 0.91 | 0.64, 1.28 | 0.87 | 0.63, 1.20 | 1.26 | 0.37, 4.38 |
| CT_858C | 1.06 | 0.79, 1.43 | 0.97 | 0.73, 1.30 | 1.79 | 0.54, 5.85 |
|  |  |  |  |  |  |  |
| ***Mycoplasma genitalium^d^*** |  |  |  |  |  |  |
| MgPaN |  |  | 0.97 | 0.73, 1.30 | 0.51 | 0.14, 1.80 |
| rMgPa |  |  | 1.04 | 0.78, 1.40 | 1.52 | 0.44, 5.26 |
|  |  |  |  |  |  |  |
| ***Human Papilloma virus*** |  |  |  |  |  |  |
| 16L1 |  |  | 0.74 | 0.39, 1.42 | 1.02 | 0.12, 8.91 |
| 18L1 |  |  | 0.89 | 0.55, 1.44 | 0.64 | 0.08, 5.31 |
|  |  |  |  |  |  |  |
| ***Polyomavirus*** |  |  |  |  |  |  |
| BKVP1 |  |  | 1.29 | 0.44, 3.82 | Could not be estimated | |
| JCVP1 |  |  | 0.60 | 0.42, 0.85 | 2.01 | 0.25, 16.21 |
| HPyV6VP1 |  |  | 1.47 | 0.86, 2.53 | Could not be estimated | |

^a^Seropositive (+)

^b^For any endometrial cancer, odds ratios are from logistic regression conditioned on matching factors (age and study site) and adjusted for oral contraceptive use, smoking, body mass index, and menopausal status. For tumor type, unconditional logistic regression adjusted for matching factors and the other variables was used.

^c^Type I tumors include those with an endometrioid, mucinous, malignant mixed Mullerian, or Mixed histologic subtype. Type II tumors include those with clear cell and papillary serous histologic subtypes.

*^d^*See manuscript Table 2 for the associations between endometrial cancer and *M. genitalium*, *Human Papilloma virus*, and *Polyomavirus*
